# Machine Learning on Mainstream Microcontrollers †

**DOI:** 10.3390/s20092638

**Published:** 2020-05-05

**Authors:** Fouad Sakr, Francesco Bellotti, Riccardo Berta, Alessandro De Gloria

**Affiliations:** Department of Electrical, Electronic and Telecommunication Engineering (DITEN)-University of Genoa, Via Opera Pia 11a, 16145 Genova, Italy; Fouad.Sakr@elios.unige.it (F.S.); riccardo.berta@unige.it (R.B.); alessandro.degloria@unige.it (A.D.G.)

**Keywords:** machine learning, edge computing, embedded devices, edge analytics, ANN, k-NN, SVM, decision trees, ARM, X-Cube-AI, STM32 Nucleo

## Abstract

This paper presents the Edge Learning Machine (ELM), a machine learning framework for edge devices, which manages the training phase on a desktop computer and performs inferences on microcontrollers. The framework implements, in a platform-independent C language, three supervised machine learning algorithms (Support Vector Machine (SVM) with a linear kernel, k-Nearest Neighbors (K-NN), and Decision Tree (DT)), and exploits STM X-Cube-AI to implement Artificial Neural Networks (ANNs) on STM32 Nucleo boards. We investigated the performance of these algorithms on six embedded boards and six datasets (four classifications and two regression). Our analysis—which aims to plug a gap in the literature—shows that the target platforms allow us to achieve the same performance score as a desktop machine, with a similar time latency. ANN performs better than the other algorithms in most cases, with no difference among the target devices. We observed that increasing the depth of an NN improves performance, up to a saturation level. k-NN performs similarly to ANN and, in one case, even better, but requires all the training sets to be kept in the inference phase, posing a significant memory demand, which can be afforded only by high-end edge devices. DT performance has a larger variance across datasets. In general, several factors impact performance in different ways across datasets. This highlights the importance of a framework like ELM, which is able to train and compare different algorithms. To support the developer community, ELM is released on an open-source basis.

## 1. Introduction

The trend of moving computation towards the edge is becoming ever more relevant, leading to performance improvements and the development of new field data processing applications [1]. This computation shift from the cloud (e.g., [2]) to the edge has advantages in terms of response latency, bandwidth occupancy, energy consumption, security and expected privacy (e.g., [3]). The huge amount, relevance and overall sensitivity of the data now collected also raise clear concerns about their use, as is being increasingly acknowledged (e.g., [4]), meaning that this is a key issue to be addressed at the societal level.

The trend towards edge computing also concerns machine learning (ML) techniques, particularly for the inference task, which is much less computationally intensive than the previous training phase. ML systems “learn” to perform tasks by considering examples, in the training phase, generally without being programmed with task-specific rules. When running ML-trained models, Internet of Things (IoT) devices can locally process their collected data, providing a prompter response and filtering the amount of bits exchanged with the cloud.

ML on the edge has attracted the interest of industry giants. Google has recently released the TensorFlow Lite platform, which provides a set of tools that enable the user to convert TensorFlow Neural Network (NN) models into a simplified and reduced version, then run this version on edge devices [5,6]. EdgeML is a Microsoft suite of ML algorithms designed to work off the grid in severely resource-constrained scenarios [7]. ARM has published an open-source library, namely Cortex Microcontroller Software Interface Standard Neural Network (CMSIS-NN), for Cortex-M processors, which maximizes NN performance [8]. Likewise, a new package, namely X-Cube-AI, has been released for implementing deep learning models on STM 32-bit microcontrollers [9].

While the literature is increasingly reporting on novel or adapted embedded machine learning algorithms, architectures and applications, there is a lack of quantitative analyses about the performance of common ML algorithms on state-of-the-art mainstream edge devices, such as ARM microcontrollers [10]. We argue that this has limited the development of new applications and the upgrading of existing ones through an edge computing extension.

In this context, we have developed the Edge Learning Machine (ELM), a framework that performs ML inference on edge devices using models created, trained, and optimized on a Desktop environment. The framework provides a platform-independent C language implementation of well-established ML algorithms, such as linear Support Vector Machine (SVM), k-Nearest Neighbors (k-NN) and Decision Tree. It also supports artificial neural networks by exploiting the X-Cube-AI package for STM 32 devices [9]. We validated the framework on a set of STM microcontrollers (families F0, F3, F4, F7, H7, and L4) using six different datasets, to answer a set of ten research questions exploring the performance of microcontrollers in typical ML Internet of Things (IoT) applications. The research questions concern a variety of aspects, ranging from inference performance comparisons (also with respect to a desktop implementation) to training time, and from pre-processing to hyperparameter tuning. The framework is released on an open-source basis (https://github.com/Edge-Learning-Machine), with a goal to support researchers in designing and deploying ML solutions on edge devices.

The remainder of this paper is organized as follows: Section 2 provides background information about the ML techniques that are discussed in the manuscript. Section 3 describes the related work in this field. Section 4 shows the implemented framework and the supported algorithms. Section 5 presents the extensive experimental analysis we conducted by exploiting the framework. Finally, Section 6 draws conclusions and briefly illustrates possible future research directions.

## 2. Background

The Edge Learning Machine framework aims to provide an extensible set of algorithms to perform inference on the edge. The current implementation features four well-established supervised learning algorithms, which we briefly introduce in the following subsections. They all support both classification and regression problems.

### 2.1. Artificial Neural Network (ANN)

An Artificial Neural Network is a model that mimics the structure of our brain’s neural network. It consists of a number of computing neurons connected to each other in a three-layer system; one input layer, several hidden layers, and one output layer. Artificial Neural Networks (ANNs) can model complex and non-linear or hidden relationships between inputs and outputs [11]. This one of the most powerful and well-known ML algorithms, which is used in a variety of applications, such as image recognition, natural language processing, forecasting, etc.

### 2.2. Linear Kernel Support Vector Machine (SVM)

The SVM algorithm is a linear classifier that computes the hyperplane that maximizes the distance from it to the nearest samples of the two target classes. It is a memory-efficient inference algorithm and is able to capture complex relationships between data points. The downside is that the training time increases with huge and noisy datasets [12]. While the algorithm deals well with non-linear problems, thanks to the utilization of kernels that map the original data in higher dimension spaces, we implemented only the original, linear kernel [13] for simplicity of implementation into the edge device.

### 2.3. K-Nearest Neighbor (k-NN)

k-NN is a very simple algorithm based on feature similarity that assigns, to a sample point, the class of the nearest set of previously labeled points. k-NN’s efficiency and performance depends on the number of neighbors K, the voting criterion (for K > 1) and the training data size. The training phase produces a very simple model (the K parameter), but the inference phase requires exploring the whole training set. Its performance is typically sensitive to noise and irrelevant features [12,14].

### 2.4. Decision Tree (DT)

This is a simple and useful algorithm, which has the advantage of clearly exposing the criteria of decisions that are made. In building the decision tree, at each step, the algorithm splits data so as to maximize the information gain, thus creating homogeneous subsets. The typical information gain criteria are Entropy and Gini. DT is able to deal with linearly inseparable data and can handle redundancy, missing values, and numerical and categorical types of data. It is negatively affected by high dimensionality and high numbers of classes, because of error propagation [12,15]. Typical hyperparameters that are tuned in the model selection phase concern regularization and typically include depth, the minimum number of samples for a leaf, and the minimum number of samples for a split, the maximum number of leaf nodes and the splitter strategy (the best one, which is the default, or a random one, which is typically used for random forests), etc. Several DTs can be randomly built for a problem, in order to create complex but high-performing random forests.

## 3. Related Work

A growing number of articles are being published on the implementation of ML on embedded systems, especially with a focus on the methodology of moving computation towards the edge. Zhang et al. [16] presented an object detector, namely MobileNet-Single Shot Detector (SSD), which was trained using a deep convolutional neural network with the popular Caffe framework. The pre-trained model was then deployed on NanoPi2, an ARM board developed by FriendlyARM, which uses Samsung Cortex-A9 Quad-Core S5P4418@1.4GHz SoC and 1 GB 32bit DDR3 RAM. MobileNet-SSD can run at 1.13FPS.

Yazici et al. [17] tested the ability of a Raspberry Pi to run ML algorithms. Three algorithms were tested, Support Vector Machine (SVM), Multi-Layer Perceptron, and Random Forests, with an accuracy above 80% and a low energy consumption. Fraunhofer Institute for Microelectronic Circuits and Systems have developed Artificial Intelligence for Embedded Systems (AIfES), a library that can run on 8-bit microcontrollers and recognize handwriting and gestures without requiring a connection to the cloud or servers [18]. Cerutti et al. [19] implemented a convolutional neural network on STM Nucleo-L476RG for people detection using CMSIS-NN, which is an optimized library that allows for the deployment of NNs on Cortex-M microcontrollers. In order to reduce the model size, weights are quantized to an 8-bit fixed point format, which slightly affects the performance. The network fits in 20 KB of flash and 6 KB of RAM with 77% accuracy. 

Google has recently released Coral Dev Board, which includes a small low power Application-Specific Integrated Circuit (ASIC) called Edge TPU, and provides high-performance ML inferencing without running the ML model on any kind of server. Edge TPU can run TensorFlow Lite, with a low processing power and high performance [20]. There are a few application programming interfaces (APIs) in the Edge tencor processing unit (TPU) module that perform inference (ClassificationEngine) for image classification, for object detection (DetectionEngine) and others that perform on-device transfer learning [21].

Microsoft is developing EdgeML, a library of machine learning algorithms that are trained on the cloud/desktop and can run on severely resource-constrained edge and endpoint IoT devices (also with 2 KB RAM), ranging from the Arduino to the Raspberry Pi [7]. They are currently releasing tree- and k-NN-based algorithms, called Bonsai and ProtoNN, respectively, for classification, regression, ranking and other common IoT tasks. Their work also concerns recurrent neural networks [22]. A major achievement concerns the translation of floating-point ML models into fixed-point code [23], which is, however, not the case in state-of-the-art mainstream microcontrollers.

The Amazon Web Services (AWS) IoT Greengrass [24] supports machine learning inference locally on edge devices. The user could use his own pre-trained model or use models that are created, trained, and optimized in Amazon SageMaker (cloud), where massive computing resources are available. AWS IoT Greengrass features lambda runtime, a message manager, resource access, etc. The minimum hardware requirements are 1 GHz of computing speed and 128 MB of RAM. 

Ghosh et al. [25] used autoencoders at the edge layer that are capable of dimensionality reduction to reduce the required processing time and storage space. The paper illustrates three scenarios. In the first one, data from sensors are sent to edge nodes, where data reduction is performed, and machine learning is then carried out in the cloud. In the second scenario, encoded data at the edge are decoded in the cloud to obtain the original amount of data and then perform machine learning tasks. Finally, pure cloud computing is performed, where data are sent from the sensors to the cloud. Results show that an autoencoder at the edge reduces the number of features and thus lowers the amount of data sent to the cloud.

Amiko’s Respiro is a smart inhaler sensor featuring an ultra-low-power ARM Cortex-M processor [26]. This sensor uses machine learning to interpret vibration data from an inhaler. The processor allows for the running of ML algorithms where the sensor is trained to recognize breathing patterns and calculate important parameters. The collected data are processed in an application and feedback is provided. 

Magno et al. [27] presented an open-source toolkit, namely FANNCortexM. It is built upon the Fast Artificial Neural Network (FANN) library and can run neural networks on the ARM Cortex-M series. This toolkit takes a neural network trained with FANN and generates code suitable for low-power microcontrollers. Another paper by Magno et al. [28] introduces a wearable multi-sensor bracelet for emotion detection that is able to run multilayer neural networks. In order to create, train, and test the neural network, the FANN library is used. To deploy the NN on the Cortex-M4F microcontroller, the above-mentioned library needs to be optimized using CMSIS and TI-Driverlib libraries. 

FidoProject is a C++ machine learning library for embedded devices and robotics [29]. It implements a neural network for classification and other algorithms such as Reinforcement Learning. Alameh et al. [30] created a smart tactile sensing system by implementing a convolutional neural network on various hardware platforms like Raspberry Pi 4, NVidia Jetson TX2, and Movidius NCS2 for tactile data decoding. 

As recent works used knowledge transfer (KT) techniques to transfer information from a large neural network to a small one in order to improve the performance of the latter, Sharma et al. [31] investigated the application of KT to edge devices, achieving good results by transferring knowledge from both the intermediate layers and the last layer of the teacher (original model) to a shallower student (target).

While most of the listed works use powerful edge devices (e.g., Cortex-A9, Raspberry PI) to test algorithms, especially NNs, there is a lack of performance analysis of common ML algorithms on mainstream microcontrollers. We intend to plug this gap by providing an open-source framework that we used for an extensive analysis.

## 4. Framework and Algorithm Understanding

**Figure 1:**
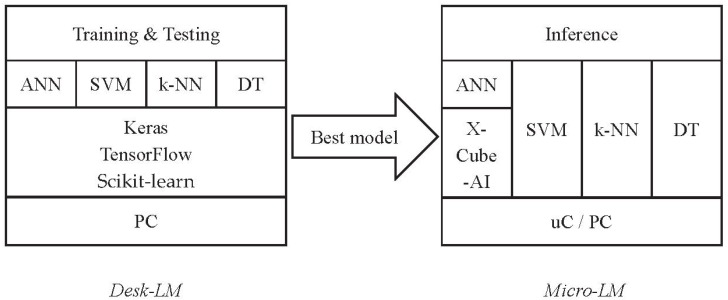
Block diagram of the Edge Learning Machine system architecture.

The proposed Edge Learning Machine (EML) framework consists of two modules, one working on the desktop (namely DeskLM, for training and testing), and one on the edge (MicroLM, for inferencing and testing), as sketched in Figure 1.

1. Desktop: the Desk-LM module is implemented in python and works on a PC to identify the best models for an input dataset. The current implementation involves four algorithms for both classification and regression: artificial neural networks (ANN), linear support vector machines (SVM), K-Nearest Neighbors (k-NN), and Decision Tree (DT) algorithms. For each algorithm, Desk-LM identifies the best model through hyperparameter tuning, as is described later in Table 2. Desk-LM relies on the scikit-learn python libraries [32] and exploits the TensorFlow [5] and Keras [33] packages for ANNs;
2. Edge: the MicroLM module reads and executes the models generated by Desk-LM. It is implemented in platform-independent C language (for linear kernel SVM, k-NN, DT) and can run on both microcontrollers and desktops, in order to perform inferences. ANNs are deployed using the X-Cube-AI expansion package for STM32 microcontrollers (TensorFlow and Keras on desktops).

**Figure 2:**
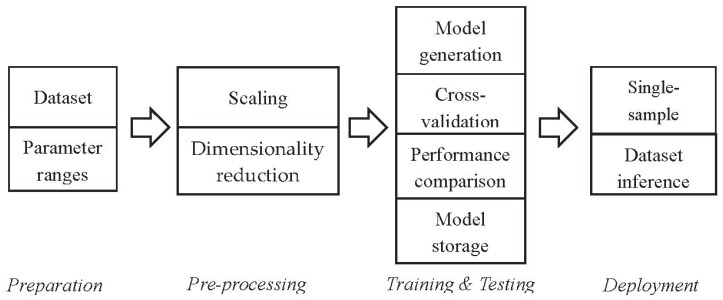
Supported workflow.

The tool has been designed to support a four-step workflow, as shown in Figure 2.

1. Preparation: in this first phase, the user provides the dataset and defines the range of the parameters to be investigated for each algorithm. The parameters are listed in Table 1 and Table 2 (common and algorithm-specific, respectively—these common parameters are used by all the algorithms, even if they have different values);
2. Preprocessing: in this phase, data goes through the scaling and dimensionality reduction steps, which are important in order to allow optimal processing by the prediction algorithms [34]. The type of algorithm used for this step is one of the common parameters set by the user (Table 1);
3. Model generation: in this phase, all the configurations resulting from combining the values of the user-specified parameters (both common and algorithm-specific; see Table 1 and Table 2, respectively) are evaluated through cross-validation, and their k values are, again, used as the parameters (Table 1). Most of the parameters (the algorithm’s hyperparameters) can be assigned a list of values, each one of which is evaluated (scikit-learn exhaustive grid search), in order to allow for the selection of the best values. At the end of this step, the best model is saved in the disk, to be deployed on the edge. Desk-LM also saves the preprocessing parameters and, if needed for performance assessment purposes, the testing set (or a reduced version of it). All these files are then compiled in Micro-LM for the processing of data on the edge;
4. Deployment: in this final phase, the MicroLM module loads the model prepared on the desktop. The deployment process for our tests on microcontrollers is done using the STM32CubeIDE integrated development environment, which exploits the X-Cube-AI pack for ANNs. The software output by our framework supports both single-sample inference and whole dataset inference, for performance analysis purposes. In the latter case, Micro-LM exploits the testing set file produced by Desk-LM.

As anticipated, the current version of the EdgeLM framework features four well-established supervised learning algorithms, of which, in the following subsections, we briefly describe the implementation on both the desktop and edge side.

### 4.1. Artificial Neural Network (ANN)

In Desk-LM, ANNs are implemented through the TensorFlow [5] and its wrapper Keras [33] packages. As an optimizer, we use adaptive moment estimation (‘adam’) [35]. At each execution run, the DeskLM module performs the hyperparameter tuning by analyzing different ranges of parameters (Table 1 and first column of Table 2) specified by the user. The ANN model hyperparameters include layer shape (number and size of input, hidden, and output layers), activation function for the hidden layers (Rectified Linear Unit (ReLU), or Tangent Activation Function (Tanh)), number of epochs, batch size, number of repeats (in order to reduce result variance), and dropout rate. The best selected model is then saved in the high-efficiency Hierarchical Data Format 5 (HDF5) compressed format [36].

For the edge implementation, DeskLM relies on the STM X-Cube-AI expansion package, which is supported by STM32CubeIDE, and allows for its integration in the application of a trained Neural Network model. The package offers the possibility of compressing models up to eight times, with an accuracy loss which is estimated by the package. The tool also provides an estimation of the complexity, through the Multiply and Accumulate Operation (MACC) figure, and of the Flash and RAM memory footprint [37].

### 4.2. Linear Support Vector Machine (SVM)

As anticipated, for the simplicity of the implementation of the edge device, we implemented only the original, linear kernel SVM [13]. The linear model executes the y = w*x + b function, where w is the support vector and b is the bias. Model selection concerns the C regularization parameter [38] (Table 2). As an output model, Desk-LM generates a C source file containing the w and b values.

### 4.3. K-Nearest Neighbor (KNN)

For simplicity of implementation, we used a Euclidean distance criterion and majority voting (for K > 1). The training phase produces a very simple model (the K parameter), but deployment also requires the availability of the whole training set (Table 2).

### 4.4. Decision Tree (DT)

In order to cope with the limited resources of edge devices, our framework allows us to analyze different tree configurations in terms of depth, leaf size, and number of splits. Concerning the splitting criterion, for simplicity of implementation on the target microcontrollers, we implemented only the “Gini” method.

## 5. Experimental Analysis and Result

We conducted the experimental analysis using six ARM Cortex-M microcontrollers produced by STM, namely F091RC, F303RE, F401RE, F746ZG, H743ZI2, and L452RE. The F series represents a wide range of microcontroller families in terms of execution time, memory size, data processing and transfer capabilities [39], while the H series provides higher performance, security, and multimedia capabilities [40]. L microcontrollers are ultra-low-power devices used in energy-efficient embedded systems and applications [41]. All listed MCUs have been used in our experiments with their STM32CubeIDE default clock values, that could be increased for a faster response. Table 3 synthesizes the main features of these devices. In the analysis, we compare the performance of the embedded devices with that of a desktop PC hosting a 2.70 GHz Core i7 processor, with 16 GB RAM and 8 MB cache.

In order to characterize the performance of the selected edge devices, we have chosen six benchmark datasets to be representative of IoT applications (Table 4). These datasets represent different application scenarios: binary classification, multiclass classification, and regression. University of California Irvine (UCI) heart disease is a popular medical dataset [42]. Virus is a dataset developed by the University of Genova to deal with data traffic analysis [43,44,45]. Sonar represents the readings of a sonar system that analyses materials, distinguishing between rocks and metallic material [46,47]. Peugeot 207 contains various parameters collected from cars, which are used to predict either the road surface or the traffic (two labels were considered in our studies: label_14: road surface and label_15: traffic) [48]. The EnviroCar dataset records various vehicular signals through the onboard diagnostic (OBDII) interface to the Controller Area Network (CAN) bus [49,50,51]. The air quality index (AQI) dataset measures air quality in Australia during a period of one year [52]. Before processing, all data were converted to float32, according to the target execution platform.

Our analysis was driven by a set of questions, synthesized in Table 5, aimed at investigating the performance of different microcontrollers in typical ML IoT contexts. We are also interested in comparing the inference performance of microcontrollers vs. desktops. The remainder of this section is devoted to the analysis of each research question. In a few cases, when the comparison is important, results are reported for every tested target platform. On the other hand, in most of the cases, when not differently stated, we chose the F401RE device as the reference for the embedded targets.

### 5.1. Performance

The first research question concerns the performance achieved both on desktop and on edge. For SVM, k-NN and DT on desktops, we report the performance of both our C implementation and the python scikit-learn implementation, while for ANN we have only the TensorFlow Keras implementation. The following set of tables show, for each algorithm, the obtained score, which is expressed in terms of accuracy (in percent, for classification problems), or coefficient of determination, R-Squared (R2, for regression problems). R2 is the proportion of the variance in the dependent variable that is predictable from the independent variable(s). The best possible score for R2 is 1.0. In scikit-learn, R2 can assume negative values, because the model can be arbitrarily worse. The second performance we consider is the inference time.

In the following (Table 6, Table 7, Table 8, Table 9, Table 10, Table 11, Table 12 and Table 13), we report two tables for each algorithm. The first one provides the best performance (in terms of score) obtained in each dataset. The second shows the hyperparameter values of the best model.

1. ANN:

Remarkably, all the embedded platforms were able to achieve the same score (accuracy or R2) as the desktop python implementation. None of the chosen datasets required the compression of the models by the STM X-Cube-AI package. ANN performed well in general, except for the Heart and Virus datasets, where the accuracy is under 90%. The inference time is relatively low in both desktop and MCUs (with similar values, in the order of ms and sometimes less). However, there is an exception in some cases—especially for Peugeot_Target_15 and Sonar—when using the F3 microcontroller.

2. Linear SVM:

As with ANN, for the linear SVM, we obtained the same score across all the target platforms, and relatively short inference times (again, with almost no difference between desktop and microcontroller implementations). However, we obtained significantly worse results than ANN for more than half of the investigated datasets. Table 9 stresses the importance of tuning the C regularization parameter, which implies the need for longer training times, particularly in the absence of normalization. We explore this in more depth when analyzing research question 9.

3. k-NN:

Notably, in some cases, the training set cap needed to be set to 100, because the Flash size was a limiting factor for some MCUs. Hence, for different training sets, we also had a different number of neighbors (K). Accordingly, the accuracy is also affected by the decrease in training set size, since the number of examples used for training is reduced. This effect is apparent for Sonar with an F0 device. This dataset has sixty features, much more than the others (typically 10–20 features). The inference time varies a lot among datasets, microcontrollers and in comparison with the desktop implementations. This is because the k-NN inference algorithm always requires the exploration of the whole training set, and thus its size plays an important role in performance, especially for less powerful devices. In the multiclass problems, k-NN exploits the larger memory availability of H7 well, outperforming SVM, and reaching a performance level close to that of ANN. It is important to highlight that the Sonar labels were reasonably well predicted by k-NN compared to ANN and SVM (92% vs. 87% and 78%). In general, k-NN achieves performance levels similar to ANN, but requires a much larger memory footprint, which is possible only on the highest-end targets.

4. DT:

When processing the EnviroCar dataset, the DT algorithm saturated the memory in most of the targets. We had to reduce the leaf size for all MCU families, apart from F7 and H7. However, this reduction did not significantly reduce the R^2^ value. In addition, DT performs worse than the others in two binary classification datasets, Heart and Sonar, and in the AQI regression dataset as well, but performs at the same level as the ANNs for the multiclass datasets and in the EnviroCar regression problem. Notably, DT achieves the fastest inference time among all algorithms, with F0 and F3 performing worse than the others, particularly in the regression problems.

As a rough summary of the first research question, we can conclude that ANN and, surprisingly, k-NN, had the highest accuracy in most cases, and Decision Tree had the shortest response time, but accuracy results were quite dependent on the dataset. The main difference between ANN and k-NN results is represented by the fact that high performance in ANN is achieved by all the targets (but not F0, which is not supported by the STM X-Cube-AI package), while k-NN poses much higher memory requirements. Concerning the timing performance, microcontrollers perform similarly to desktop implementations on the studied datasets. The only exception is found in k-NN, for which each inference requires the exploration of the whole dataset, and the corresponding computational demand penalizes the performance, especially on low-end devices. When comparing the edge devices, the best time performance is achieved by F7 and H7 (and we used default clock speeds, that can be significantly increased). Unsurprisingly, given the available hardware, F0 performs worse than all the others. Considering the score, we managed to train all the edge devices to achieve the same level of performance as the desktop in each algorithm, with the exception of k-NN in the multiclass tests (Peugeot), where only H7 is able to perform like a desktop, but with a significant time performance penalty. On the other hand, F0 performs significantly worse than the other edge devices in the k-NN Sonar binary classification.

### 5.2. Scaling

Feature preprocessing is applied to the original features before the training phase, with the goal of increasing prediction accuracy and speeding up response times [34]. Since the range of values is typically different from one feature to another, the proper computation of the objective function requires normalized inputs. For instance, the computation of the Euclidean distance between points is governed by features with a broader value range. Moreover, gradient descent converges much faster on normalized values [53].

We considered three cases that we applied on ANN, SVM, and k-NN: no scaling, MinMax Scaler, and Standard Scaler (Std) [54]. The set of tables below (Table 14, Table 15, Table 16, Table 17 and Table 18) show the accuracy of R^2^ for all datasets under various scaling conditions. Most common DT algorithms are invariant to monotonic transformations [55], so we did not consider DT in this analysis.

1. ANN:

2. SVM:

3. k-NN:

These results clearly show the importance across all the datasets and algorithms of scaling the inputs. For instance, MinMax scaling allowed ANNs to reach 99% accuracy in Virus (from a 74% baseline), and Peugeot 14 (from 95%) and 0.86 R2 (from 0.70) in AQI. The application of MinMax allowed SVM to achieve 94% accuracy in Virus (form 71%) and 91% accuracy in Peugeot 14 (from 50%). Standard input scaling improved the k-NN accuracy of Heart from 63% to 83%. For large regression datasets, especially with SVM (see also research question 9), input scaling avoids large training times.

### 5.3. Principal Component Analysis (PCA)

Dimensionality reduction allows us to reduce the effects of noise, space and processing requirements. One well-known method is Principal Component Analysis (PCA), which performs an orthogonal transformation to convert a set of observations of possibly correlated variables into a set of values of linearly independent variables, which are called principal components [24]. We tried different values of PCA dimension reduction: none, 30% (i.e., the algorithm selects a number of components such that the amount of variance that needs to be explained is greater than 30%), and automatic maximum likelihood estimation (mle) [56], whose results are shown in Table 19, Table 20, Table 21, Table 22, Table 23, Table 24, Table 25 and Table 26.

1. SVM:

2. k-NN:

3. ANN:

4. DT:

The results reported in the above tables are quite varied. The 30% PCA value is frequently too low, except for the Sonar dataset, which has 60 features, much more than the others, and thus looks less sensitive to such a coarse reduction. For SVM, PCA does not perform better than or equal to mle, while the opposite is true for k-NN. Moreover, in ANNs, mle tends to provide better results, except AQI. In DT, there is a variance of outcomes. Mle does not perform any better than the other algorithms in Heart (78% vs. 67% accuracy) and Sonar (76% vs. 65%), while performance decreases for Peugeot 14 (93% vs. 99%) and AQI (0.49 vs. 0.65). For AQI, PCA never improves performance. The opposite is true for Heart and (except SVM) Sonar.

### 5.4. ANN Layer Configuration

To answer this question, we investigated performance among four ANN hidden-layer configurations, as follows:

1. One hidden layer of 50 neurons;
2. One hidden layer of 500 neurons;
3. Three hidden layers of 100 neurons each;
4. Four hidden layers with 300, 200, 100, 50 neurons, respectively.

Table 27, Table 28 and Table 29 indicate the highest performance for each layer shape.

By observing the results, we can see that deepening the network tends to improve the results, but only up to a certain threshold. For the Heart dataset, which has the lowest overall accuracy, we tried additional, deeper shapes beyond those reported in Table 27, Table 28 and Table 29, but with no better results. On the other hand, widening the first layer provides only slightly better results (and in one case worsens them).

### 5.5. ANN Activation Function

Another relevant design choice concerns the activation function in the hidden layers. Activation functions are attached to each neuron in the network and define its output. They introduce a non-linear factor in the processing of a neural network. Two activation functions are typically used: Rectified Linear Unit (ReLU) and Tangent Activation Function (Tanh). On the other hand, for the output layer, we used a sigmoid for binary classification models as an activation function, and a softmax for multiclassification tasks. For regression problems, we created an output layer without any activation function (i.e., we use the default “linear” activation), as we are interested in predicting numerical values directly, without transformation. Table 30 and Table 31 show the highest accuracy achieved in hidden layers for each function, alongside its corresponding configuration.

The results are similar, with a slight prevalence of ReLU, with a valuable difference for Sonar (+7% accuracy) and AQI (+5% R2).

### 5.6. ANN Batch Size

The batch size is the number of training examples processed in one iteration before the model being trained is updated. To test the effect of this parameter, we considered three values, one, 10, and 20, keeping the number of epochs fixed to 20. Table 32 shows the accuracy of each dataset for various batch sizes.

The results show that the value of 10 provides optimal results in terms of accuracy. Actually, the difference becomes relevant only for the case of AQI. A batch size equal to one poses an excessive time overhead (approximately 30% slower than the batch size of 10), while a batch size of 20 achieves a speedup of about 40%.

### 5.7. ANN Accuracy vs. Epochs

**Figure 3:**
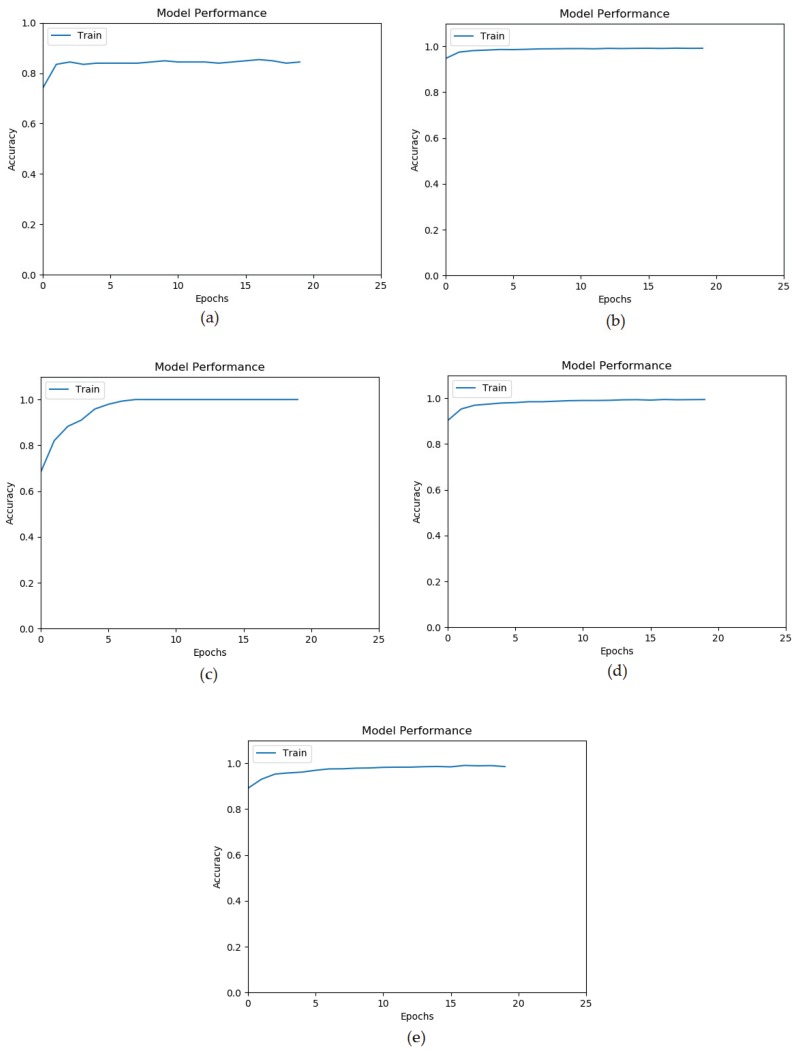
Accuracy vs. Epochs for (**a**) Heart, (**b**) Virus, (**c**) Sonar, (**d**) Peugeot target 14, and (**e**) Peugeot target 15.

**Figure 4:**
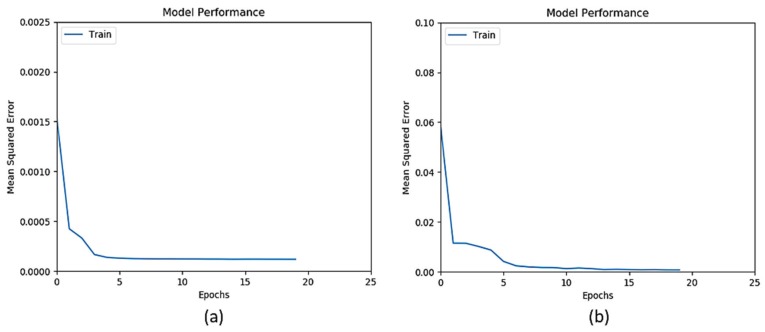
Mean Squared Error vs. Epochs for (**a**) EnviroCar, and (**b**) air quality index (AQI).

ANN training goes through several epochs, where an epoch is a learning cycle in which the learner model sees the whole training data set. Figure 3 and Figure 4 show that the training of ANN on all datasets converges quickly within 10 epochs.

### 5.8. ANN Dropout

Dropout is a simple method to prevent overfitting in ANNs. It consists of randomly ignoring a certain number of neuron outputs in a layer during the training phase.

The results in Table 33 show that this regularization step provides no improvement in the considered cases, but has a slight negative effect in a couple of datasets (Sonar and AQI).

### 5.9. SVM Regularization Training Time

In SVM, *C* is a key regularization parameter, that controls the tradeoff between errors of the SVM on training data and margin maximization [13,57]. The classification rate is highly dependent on this coefficient, as confirmed by Table 8 and Table 9. Desk-LM uses the grid search method to explore the C values presented by the user, which require long waiting times in some cases. To quantify this, we measured the training latency time in a set of typical values (*C* = 0.01, 0.1, 1, 10, and 100), with the results provided in Table 34.

Different values of the *C* parameter have an impact on the training time. The table shows that higher *C* values require higher training time. We must stress that the above results represent the training time for the best models. In particular, when no normalization procedure was applied, the training time using large values of C became huge (also up to one hour), especially for regression datasets.

### 5.10. DT Parameters

**Figure 5:**
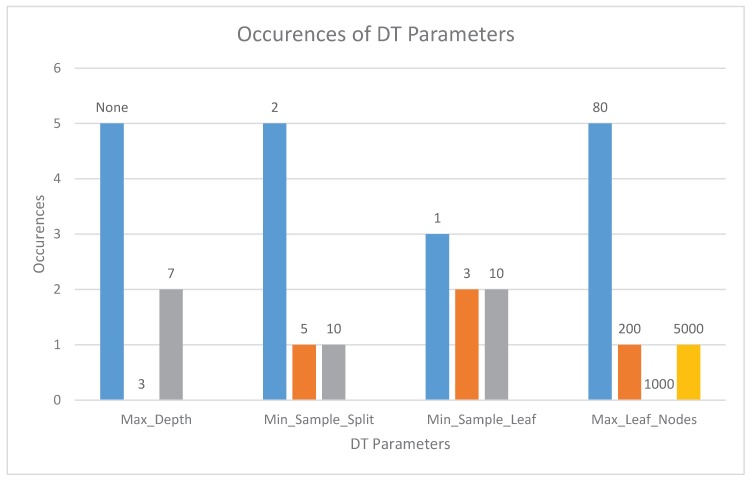
Number of occurrences of each DT parameter.

Tuning a decision tree requires us to test the effect of various hyperparameters, such as *max_depth*, *min_simple_split*. Figure 5 shows the distribution of the tested parameter values for the best models in the different datasets (see also Table 13 to see the best results).

In most cases, the whole tree depth is needed, and this does not exceed the memory available in the microcontrollers. However, *Max_Leaf_Nodes* values usually need a low threshold (80). EnviroCar required a high value of 5000, which had to be reduced down to 1000 for F3, F4, L4 and to 200 for F0 because of the limited RAM availability.

## 6. Conclusions and Future Work

This paper presented the Edge Learning Machine (ELM), a machine learning platform for edge devices. ELM performs training on desktop computers, exploiting TensorFlow, Keras, and scikit-learn, and makes inferences on microcontrollers. It implements, in platform-independent C language, three supervised machine learning algorithms (Linear SVM, k-NN, and DT), and exploits the STM X-Cube-AI package for implementing ANNs on STM32 Nucleo boards. The training phase on Desk-LM searches for the best configuration across a variety of user-defined parameter values. In order to investigate the performance of these algorithms on the targeted devices, we posed ten research questions (RQ 1–10, in the following) and analyzed a set of six datasets (four classifications and two regressions). To the best of our knowledge, this is the first paper presenting such an extensive performance analysis of edge machine learning in terms of datasets, algorithms, configurations, and types of devices.

Our analysis shows that, on a set of available IoT data, we managed to train all the targeted devices to achieve, with at least one algorithm, the best score (classification accuracy or regression R2) obtained through a desktop machine (RQ1). ANN performs better than the other algorithms in most of the cases, without differences among the target devices (apart from F0, that is not supported by STM X-Cube-AI). k-NN performs similarly to ANN, and in one case even better, but requires that all the training sets are kept in the inference phase, posing a significant memory demand, which penalizes time performance, particularly on low-end devices. The performance of Decision Tree performance varied widely across datasets. When comparing edge devices, the best time performance is achieved by F7 and H7. Unsurprisingly, given the available hardware, F0 performs worse than all the others.

The preprocessing phase is extremely important. Results across all the datasets and algorithms show the importance of scaling the inputs, which lead to improvements of up to 82% in accuracy (SVM Virus) and 23% in R2 (k-NN Heart) (RQ2). The applications of PCA have various effects across algorithms and datasets (RQ3).

In terms of the ANN hyperparameters, we observed that increasing the depth of a NN typically improves its performance, up to a saturation level (RQ4). When comparing the neuron activation functions, we observed a slight prevalence of ReLU over Tanh (RQ5). The batch size has little influence on score, but it does have an influence on training time. We established that 10 was the optimal value for all the examined datasets (RQ6). In all datasets, the ANN training quickly converges within 10 epochs (RQ7). The dropout regularization parameter only led to some slight worsening in a couple of datasets (RQ8).

In SVM, the C hyperparameter value selection has an impact on training times, but only when inputs are not scaled (RQ9). In most datasets, the whole tree depth is needed for DT models, and this does not exceed the memory available in the microcontrollers. However, the values of *Max_Leaf_Nodes* usually require a low threshold value (80) (RQ10).

As synthesized above, in general, several factors impact performance in different ways across datasets. This highlights the importance of a framework like ELM, which is able to test different algorithms, each one with different configurations. To support the developer community, ELM is released on an open-source basis.

As a possible direction for future work, we consider that the analysis should be extended to include different types of NNs (Convolutional Neural Networks, Recurrent Neural Networks) with more complex datasets (e.g., also including images and audio streams). An extensive analysis should also be performed on unsupervised algorithms that look particularly suited for immediate field deployment, especially in low-accessibility areas. As the complexity of IoT applications is likely to increase, we also expect that distributed ML at the edge will probably be a significant challenge in the coming years.

## Figures and Tables

**Table 1:** Common configuration parameters.

Common Parameters
Algorithm type (SVM, k-NN, DT, ANN)
Dataset
Content format (dataset start and end column, target column, etc.)
Number of classes (if classification)
Testing set size
Regression (True or False)
PCA (a specific number of features or MLE algorithm)
Normalization (standard or minmax)
K-fold cross-validation
Scoring metrics (accuracy, R2)

**Table 2:** Algorithm-specific configuration parameters.

Algorithm-Specific Configuration			
ANN	Linear SVM	k-NN	DT
Layer Shape	C	K (number of neighbors)	Splitting criterion
Activation Function		Training set size for targets	max_depth
Dropout			min_samples_split
Loss metrics			min_samples_leaf
Number of epochs			max_leaf_nodes
Batch size			
Number of repeats			

**Table 3:** Microcontroller specifications.

Microcontroller	Flash Memory	SRAM	Processor Speed Used (MHz)	Processor Cost ($)	Board Cost ($)
F091RC	256 Kb	32 Kb	48 (max: 48)	4.8	10.32
F303RE	512 Kb	80 Kb	72 (max: 72)	7.72	10.32
F401RE	512 Kb	96 Kb	84 (max: 84)	6.43	13
F746ZG	1 Mb	340 Kb	96 (max: 216)	12.99	23
H743ZI2	2 Mb	1 Mb	96 (max: 480)	13.32	27
L452RE	512 Kb	160 Kb	80 (max: 80)	7.03	14

**Table 4:** Dataset specifications.

Dataset	Samples Features	Type
Heart	303 × 13	Binary Classification
Virus	24736 × 13	Binary Classification
Sonar	209 × 60	Binary Classification
Peugeot 207 *	8615 × 14	Multiclass Classification
EnviroCar	47077 × 5	Regression
AQI	367 × 8	Regression

* For Peugeot 207, we considered two different labels.

**Table 5:** Research questions.

Research Questions (RQ)	Description
1. Performance	Score (accuracy, R2) and inference time
2. Scaling	Effect of scaling data
3. PCA	Effect of dimensionality reduction on score
4. ANN Layer Configuration	Different layer shapes (depth and thickness)
5. ANN Activation Function	Effect of different neuron activation functions
6. ANN Batch Size	Effect of batch size on score and time
7. ANN Epochs	Effect of number of epochs in training
8. ANN Dropout	Effect of a regularization technique to avoid overfitting
9. SVM Regularization Training Time	SVM training time with different values of the “C” regularization parameter
10. DT Parameters	Tuning the decision tree

**Table 6:** Artificial Neural Network (ANN) performance.

ANN									
DatasetDataset		Performance	Desktop	MCUs					
Type	Name		Python	F0	F3	F4	F7	H7	L4
Binary	Heart	Accuracy	84%	*	84%	84%	84%	84%	84%
		Inf. Time	<1 ms	*	3 ms	1 ms	<1 ms	<1 ms	1 ms
	Virus	Accuracy	99%	*	99%	99%	99%	99%	99%
		Inf. Time	<1 ms	*	5 ms	3 ms	1 ms	1 ms	4 ms
	Sonar	Accuracy	87%	*	87%	87%	87%	87%	87%
		Inf. Time	<1 ms	*	16 ms	8 ms	3 ms	3 ms	10 ms
Multiclass	Peugeot_Target 14	Accuracy	99%	*	99%	99%	99%	99%	99%
		Inf. Time	<1 ms	*	2 ms	1 ms	<1 ms	<1 ms	1 ms
	Peugeot_Target 15	Accuracy	99%	*	99%	99%	99%	99%	99%
		Inf. Time	<1 ms	*	18 ms	10 ms	4 ms	4 ms	12 ms
Regression	Enviro Car	R^2^	0.99	*	0.99	0.99	0.99	0.99	0.99
		Inf. Time	<1 ms	*	<1 ms	<1 ms	<1 ms	<1 ms	<1 ms
	AQI	R^2^	0.86	*	0.86	0.86	0.86	0.86	0.86
		Inf. Time	<1 ms	*	4 ms	2 ms	1 ms	1 ms	3 ms

*: F0 not supported by the STM X-Cube-AI package.

**Table 7:** ANN corresponding configurations.

Dataset	Best Configuration (Table 1, Table 2)				
	AF	LC	PCA	Dropout	Scaling
Heart	Tanh	[500]	30%	0	StandardScaler
Virus	Tanh	[100,100,100]	None	0	StandardScaler
Sonar	ReLU	[300,200,100,50]	30%	0	MinMaxScaler
Peugeot_Target 14	ReLU	[500]	None	0	StandardScaler
Peugeot_Target 15	Tanh	[300,200,100,50]	None	0	StandardScaler
EnviroCar	Tanh	[50]	mle	0	MinMaxScaler
AQI	ReLU	[300,200,100,50]	None	0	MinMaxScaler

Activation Function (AF), Layer Configuration (LC), Principal Component Analysis (PCA).

**Table 8:** Linear Support Vector Machine (SVM) performance.

Linear SVM										
Dataset		Score	Desktop		MCUs					
Type	Name		Python	C	F0	F3	F4	F7	H7	L4
Binary	Heart	Acc.	84%	84%	84%	84%	84%	84%	84%	84%
		Inf. Time	<1 ms	<1 ms	<1 ms	<1 ms	<1 ms	<1 ms	<1 ms	<1 ms
	Virus	Acc.	94%	94%	94%	94%	94%	94%	94%	94%
		Inf. Time	<1 ms	<1 ms	1 ms	<1 ms	<1 ms	<1 ms	<1 ms	<1 ms
	Sonar	Acc.	78%	78%	78%	78%	78%	78%	78%	78%
		Inf. Time	<1 ms	<1 ms	3 ms	<1 ms	<1 ms	<1 ms	<1 ms	<1 ms
Multiclass	Peugeot_Target 14	Acc.	91%	91%	91%	91%	91%	91%	91%	91%
		Inf. Time	<1 ms	<1 ms	2 ms	<1 ms	<1 ms	<1 ms	<1 ms	<1 ms
	Peugeot_Target 15	Acc.	90%	90%	90%	90%	90%	90%	90%	90%
		Inf. Time	<1 ms	<1 ms	2 ms	<1 ms	<1 ms	<1 ms	<1 ms	<1 ms
Regress	EnviroCar	R^2^	0.99	0.99	0.99	0.99	0.99	0.99	0.99	0.99
		Inf. Time	<1 ms	<1 ms	5 ms	3 ms	<1 ms	<1 ms	<1 ms	<1 ms
	AQI	R^2^	0.73	0.73	0.73	0.73	0.73	0.73	0.73	0.73
		Inf. Time	<1 ms	<1 ms	5 ms	3 ms	<1 ms	<1 ms	<1 ms	<1 ms

**Table 9:** Linear SVM corresponding configuration.

Dataset	Best Configuration (Table 1, Table 2)		
	C	PCA	Scaling
Heart	0.1	30%	StandardScaler
Virus	1	None	StandardScaler
Sonar	0.01	None	StandardScaler
Peugeot_Target 14	0.1	mle	StandardScaler
Peugeot_Target 15	10	mle	StandardScaler
EnviroCar	0.1	None	StandardScaler
AQI	1	mle	StandardScaler

SVM regularization parameter (C), Principal Component Analysis (PCA).

**Table 10:** k-Nearest Neighbors (k-NN) performance.

k-NN										
Dataset		Score	Desktop		MCUs					
Type	Name		Python	C	F0	F3	F4	F7	H7	L4
Bin.	Heart	Acc.	83%	83%	83%	83%	83%	83%	83%	83%
		Inf Time	<1 ms	<1 ms	366 ms	71 ms	7 ms	4 ms	4 ms	8 ms
	Virus	Acc.	99%	99%	95%	95%	95%	95%	95%	95%
		Inf Time	<1 ms	<1 ms	199 ms	38 ms	4 ms	3 ms	3 ms	4 ms
	Sonar	Acc.	92%	92%	76%	92%	92%	92%	92%	92%
		Inf Time	<1 ms	<1 ms	329 ms	140 ms	14 ms	10 ms	10 ms	14 ms
Multi class	Peugeot_Target 14	Acc.	98%	98%	88%	88%	88%	88%	98%	88%
		Inf Time	<1 ms	<1 ms	205 ms	39 ms	4 ms	3 ms	200 ms	4 ms
	Peugeot_Target 15	Acc.	97%	97%	86%	86%	86%	86%	97%	86%
		Inf Time	<1 ms	<1 ms	204 ms	39 ms	4 ms	3 ms	200 ms	4 ms
Regr	Enviro Car	R^2^	0.99	0.99	0.97	0.97	0.97	0.97	0.97	0.97
		Inf Time	<1 ms	<1 ms	125 ms	30 ms	3 ms	2 ms	2 ms	4 ms
	AQI	R^2^	0.73	0.73	0.73	0.73	0.73	0.73	0.73	0.73
		Inf Time	<1 ms	<1 ms	414 ms	85 ms	9 ms	6 ms	6 ms	10 ms

**Table 11:** k-NN corresponding configurations.

Dataset	Best Configuration (Table 1, Table 2)			
	K	PCA	Scaling	Notes
Heart	10	mle	StandardScaler	This configuration fits all targets
Virus	1	None	StandardScaler	In all MCUs K = 1, training set cap = 100
Sonar	1	None	MinMaxScaler	In F0 K = 1, training set cap = 50
Peugeot_Target 14	1	mle	MinMaxScaler	In F0, F3, F4, F7, L4 K = 4, training set cap = 100
Peugeot_Target 15	1	mle	MinMaxScaler	In F0, F3, F4, F7, L4 K = 3, training set cap = 100
EnviroCar	3	mle	MinMaxScaler	In all MCUs K = 2, training set cap = 100
AQI	1	mle	StandardScaler	This configuration fits all targets

Number of neighbors (K), Principal Component Analysis (PCA).

**Table 12:** Desktop (DT) performance.

DT										
Dataset		Score	Desktop		MCUs					
Type	Name		Python	C	F0	F3	F4	F7	H7	L4
Bin.	Heart	Accuracy	78%	78%	78%	78%	78%	78%	78%	78%
		Inf. Time	<1 ms	<1	<1	<1	<1	<1	<1	<1
	Virus	Accuracy	99%	99%	99%	99%	99%	99%	99%	99%
		Inf. Time	<1 ms	<1	<1	<1	<1	<1	<1	<1
	Sonar	Accuracy	76%	76%	76%	76%	76%	76%	76%	76%
		Inf. Time	<1 ms	<1	<1	<1	<1	<1	<1	<1
Multi class	Peugeot_Target 14	Accuracy	99%	99%	99%	99%	99%	99%	99%	99%
		Inf. Time	<1 ms	<1	<1	<1	<1	<1	<1	<1
	Peugeot_Target 15	Accuracy	98%	98%	98%	98%	98%	98%	98%	98%
		Inf. Time	<1 ms	<1	<1	<1	<1	<1	<1	<1
Regr	Enviro Car	R^2^	0.99	0.99	0.99	0.99	0.99	0.99	0.99	0.99
		Inf. Time	<1	<1	2	2	<1	<1	<1	<1
	AQI	R^2^	0.65	0.65	0.65	0.65	0.65	0.65	0.65	0.65
		Inf. Time	<1 ms	<1	2	2	<1	<1	<1	<1

**Table 13:** DT corresponding configurations. Time is in ms (omitted for reasons of space).

Dataset	Best Configuration (Table 1, Table 2)					
	Max Depth	Min Sample Split	Min Sample Leaf	Max Leaf Nodes	PCA	Notes
Heart	7	2	10	80	mle	*
Virus	None	5	1	80	None	*
Sonar	7	2	10	80	mle	*
Peugeot_Target 14	None	2	3	80	None	*
Peugeot_Target 15	None	2	1	200	None	*
EnviroCar	None	2	1	5000	None	Max leaf nodes = 1000 in F3, F4, L4; max leaf nodes = 200 for F0
AQI	None	10	3	80	None	*

Principal Component Analysis (PCA). * This configuration fits all targets.

**Table 14:** Performance and configuration of ANN with no scaling.

ANN				
Dataset	None	Configuration		
		AF	LC	PCA
Heart	78%	ReLU	[500]	mle
Virus	74%	Tanh	[100, 100, 100]	mle
Sonar	85%	ReLU	[100, 100, 100]	mle
Peugeot_Target 14	95%	Tanh	[500]	mle
Peugeot_Target 15	92%	ReLU	[100, 100, 100]	mle
EnviroCar	0.97	Tanh	[50]	mle
AQI	0.70	ReLU	[300, 200, 100, 50]	None

Activation Function (AF), Layer Configuration (LC), Principal Component Analysis (PCA).

**Table 15:** Performance and configuration of ANN with MinMax scaling.

ANN				
Dataset	MinMax	Configuration		
		AF	LC	PCA
Heart	80%	Tanh	[300, 200, 100, 50]	30%
Virus	99%	ReLU	[100, 100, 100]	None
Sonar	87%	ReLU	[300, 200, 100, 50]	30%
Peugeot_Target 14	99%	ReLU	[100, 100, 100]	mle
Peugeot_Target 15	98%	Tanh	[300, 200, 100, 50]	mle
EnviroCar	0.99	ReLU	[50]	mle
AQI	0.86	ReLU	[300, 200, 100, 50]	None

Activation Function (AF), Layer Configuration (LC), Principal Component Analysis (PCA).

**Table 16:** Performance and configuration of ANN with StandardScaler normalization.

ANN				
Dataset	Std	Configuration		
		AF	LC	PCA
Heart	84%	Tanh	[500]	30%
Virus	99%	Tanh	[100, 100, 100]	None
Sonar	86%	ReLU	[100, 100, 100]	mle
Peugeot_Target 14	99%	ReLU	[500]	None
Peugeot_Target 15	99%	Tanh	[300, 200, 100, 50]	None
EnviroCar	0.99	Tanh	[50]	mle
AQI	0.84	ReLU	[300, 200, 100, 50]	None

Activation Function (AF), Layer Configuration (LC), Principal Component Analysis (PCA).

**Table 17:** Performance and configuration of SVM for different scaling techniques.

SVM									
Dataset	None	Configuration		MinMax	Configuration		Std	Configuration	
		C	PCA		C	PCA		C	PCA
Heart	78%	0.01	mle	79%	1	mle	84%	0.1	30%
Virus	71%	0.01	mle	94%	100	None	94%	1	None
Sonar	77%	0.1	mle	73%	0.1	None	78%	0.01	None
Peugeot_Target 14	50%	0.1	mle	91%	10	mle	91%	0.1	mle
Peugeot_Target 15	76%	0.01	mle	90%	10	mle	90%	10	mle
EnviroCar	0.97	Slow	mle	0.98	0.1	None	0.99	0.1	None
AQI	0.7	100	mle	0.62	100	30%	0.73	1	mle

SVM regularization parameter (C), Principal Component Analysis (PCA).

**Table 18:** Performance and configuration of k-NN for different scaling techniques.

k-NN									
Dataset	None	Configuration		MinMax	Configuration		Std	Configuration	
		K	PCA		K	PCA		K	PCA
Heart	63%	3	mle	75%	3	None	83%	10	mle
Virus	95%	1	mle	99%	1	None	99%	1	None
Sonar	77%	1	mle	92%	1	None	87%	1	None
Peugeot_Target 14	91%	1	mle	98%	1	mle	97%	1	mle
Peugeot_Target 15	89%	2	mle	97%	1	mle	97%	1	None
EnviroCar	0.98	5	mle	0.99	3	mle	0.99	3	None
AQI	0.73	4	mle	0.7	3	mle	0.73	6	mle

Number of neighbors (K), Principal Component Analysis (PCA).

**Table 19:** SVM performance and configuration for various PCA values.

SVM									
Dataset	PCA = None			PCA = 30%			PCA = mle		
	Score	Configuration		Score	Configuration		Score	Configuration	
		C	Scaling		C	Scaling		C	Scaling
Heart	78%	0.01	Std	84%	0.1	Std	79%	0.1	Std
Virus	99%	1	Std	86%	100	MinMax	94%	0.1	Std
Sonar	78%	0.01	Std	75%	100	Std	77%	0.01	Std
Peugeot_Target 14	91%	10	MinMax	83%	0.1	MinMax	91%	0.1	Std
Peugeot_Target 15	90%	10	MinMax	86%	10	MinMax	90%	10	Std
EnviroCar	0.99	0.1	Std	0.94	0.1	MinMax	0.98	0.1	MinMax
AQI	0.73	10	Std	0.71	100	Std	0.73	1	Std

SVM regularization parameter (C).

**Table 20:** k-NN performance and configuration for various PCA techniques.

k-NN									
Dataset	PCA = None			PCA = 30%			PCA = mle		
	Score	Configuration		Score	Configuration		Score	Configuration	
		K	Scaling		K	Scaling		K	Scaling
Heart	77%	3	Std	76%	13	Std	83%	10	Std
Virus	99%	1	Std	99%	1	Std	99%	1	Std
Sonar	92%	1	MinMax	84%	1	MinMax	97%	1	Std
Peugeot Targ 14	98%	1	MinMax	92%	3	MinMax	98%	1	Std
Peugeot Targ 15	97%	1	MinMax	90%	6	MinMax	97%	1	Std
EnviroCar	0.99	3	MinMax	0.99	9	Std	0.99	3	MinMax
AQI	0.73	6	Std	0.57	3	MinMax	0.73	6	Std

Number of neighbors (K).

**Table 21:** ANN performance and configuration for PCA = None.

ANN				
Dataset	Score	Configuration		
		AF	LC	Scaling
Heart	81%	Tanh	[100, 100, 100]	Std
Virus	99%	Tanh	[100, 100, 100]	Std
Sonar	84%	ReLU	[50]	Std
Peugeot_Target 14	99%	ReLU	[500]	Std
Peugeot_Target 15	99%	Tanh	[300, 200, 100, 50]	Std
EnviroCar	0.99	Tanh	[50]	MinMax
AQI	0.86	ReLU	[300,200,100,50]	MinMax

Activation Function (AF), Layer Configuration (LC).

**Table 22:** ANN performance and configuration for PCA = 30%.

ANN				
Dataset	Score	Configuration		
		AF	LC	Scaling
Heart	84%	Tanh	[500]	Std
Virus	98%	ReLU	[100, 100, 100]	Std
Sonar	87%	ReLU	[300, 200, 100, 50]	MinMax
Peugeot_Target 14	92%	ReLU	[50]	MinMax
Peugeot_Target 15	90%	ReLU	[50]	MinMax
EnviroCar	0.98	ReLU	[50]	MinMax
AQI	0.71	ReLU	[100,100,100]	MinMax

Activation Function (AF), Layer Configuration (LC).

**Table 23:** ANN performance and configuration for PCA = mle.

ANN				
Dataset	Score	Configuration		
		AF	LC	Scaling
Heart	83%	Tanh	[300,200,100,50]	Std
Virus	99%	Tanh	[100,100,100]	Std
Sonar	86%	ReLU	[100,100,100]	Std
Peugeot_Target 14	99%	Tanh	[100,100,100]	Std
Peugeot_Target 15	99%	Tanh	[300,200,100,50]	Std
EnviroCar	0.99	Tanh	[50]	MinMax
AQI	0.82	Tanh	[300,200,100,50]	MinMax

Activation Function (AF), Layer Configuration (LC).

**Table 24:** DT performance and configuration for PCA = None.

DT					
Dataset	Score	Configuration			
		Max-Depth	Min_Sample_Split	Min_Sample_Leaf	Max_Leaf_Nodes
Heart	67%	7	2	10	80
Virus	99%	None	5	1	80
Sonar	65%	7	2	10	80
Peugeot_Target 14	99%	None	2	3	80
Peugeot_Target 15	98%	None	2	1	200
EnviroCar	0.99	None	2	1	5000
AQI	0.65	None	10	3	80

**Table 25:** DT performance and configuration for PCA = 30%.

DT					
Dataset	Score	Configuration			
		Max-Depth	Min_Sample_Split	Min_Sample_Leaf	Max_Leaf_Nodes
Heart	62%	3	2	1	80
Virus	96%	None	2	1	1000
Sonar	75%	7	2	3	80
Peugeot_Target 14	84%	None	2	1	200
Peugeot_Target 15	87%	7	2	10	80
EnviroCar	0.98	None	2	10	1000
AQI	0.62	7	10	1	80

**Table 26:** DT performance and configuration for PCA = mle.

DT					
Dataset	Score	Configuration			
		Max-Depth	Min_Sample_Split	Min_Sample_Leaf	Max_Leaf_Nodes
Heart	78%	7	2	10	80
Virus	99%	None	2	1	200
Sonar	76%	7	2	10	80
Peugeot_Target 14	93%	None	5	1	80
Peugeot_Target 15	92%	None	10	1	80
EnviroCar	0.98	None	2	10	5000
AQI	0.49	7	5	1	80

**Table 27:** Results for layer configuration LC = [50] and LC = [500]. We have omitted columns with all zero Dropout values.

Dataset	LC = [50]	Configuration			LC = [500]	Configuration		
		AF	PCA	Scaling		AF	PCA	Scaling
Heart	83%	Tanh	30%	Std	84%	Tanh	30%	Std
Virus	98%	ReLU	None	Std	98%	ReLU	None	Std
Sonar	84%	ReLU	None	Std	81%	ReLU	mle	Std
Peugeot_Target 14	98%	ReLU	mle	Std	99%	ReLU	None	Std
Peugeot_Target 15	98%	ReLU	mle	Std	98%	ReLU	None	Std
EnviroCar	0.99	Tanh	mle	MinMax	0.99	ReLU	mle	MinMax
AQI	0.76	Tanh	mle	MinMax	0.78	ReLU	mle	MinMax

Activation Function (AF), Principal Component Analysis (PCA).

**Table 28:** Layer configuration LC = [100,100,100] results.

Dataset	LC = [100,100,100]	Configuration			
		AF	PCA	Scaling	Dropout
Heart	84%	Tanh	30%	Std	0
Virus	99%	Tanh	None	Std	0
Sonar	87%	ReLU	30%	Std	0
Peugeot Target 14	99%	ReLU	mle	Std	0
Peugeot Target 15	98%	Tanh	mle	Std	0.1
EnviroCar	0.99	Tanh	mle	MinMax	0
AQI	0.80	ReLU	mle	MinMax	0

Activation Function (AF), Principal Component Analysis (PCA).

**Table 29:** Layer Configuration (LC) = [300,200,100,50] results.

Dataset	LC = [300,200,100,50]	Configuration		
		AF	PCA	Scaling
Heart	84%	Tanh	30%	Std
Virus	99%	ReLU	None	Std
Sonar	87%	ReLU	30%	MinMax
Peugeot Target 14	99%	ReLU	mle	MinMax
Peugeot Target 15	99%	Tanh	None	Std
EnviroCar	0.99	Tanh	mle	MinMax
AQI	0.86	ReLU	None	MinMax

Activation Function (AF), Principal Component Analysis (PCA).

**Table 30:** ReLU activation function results.

Dataset	Score	Best Configuration		
		LC	PCA	Scaling
Heart	83%	[500]	30%	Std
Virus	99%	[100,100,100]	mle	Std
Sonar	87%	[300,200,100,50]	30%	MinMax
Peugeot_Target 14	99%	[500]	None	Std
Peugeot_Target 15	99%	[300,200,100,50]	None	Std
EnviroCar	0.99	[50]	mle	MinMax
AQI	0.86	[300,200,100,50]	None	MinMax

Layer Configuration (LC), Principal Component Analysis (PCA).

**Table 31:** Tanh activation function results.

Dataset	Score	Best Configuration			
		LC	PCA	Scaling	Dropout
Heart	84%	[500]	30%	Std	0
Virus	99%	[100,100,100]	None	Std	0
Sonar	81%	[50]	30%	MinMax	0
Peugeot_Target 14	99%	[100,100,100]	mle	Std	0
Peugeot_Target 15	99%	[300,200,100,50]	None	Std	0
EnviroCar	0.99	[50]	mle	MinMax	0
AQI	0.82	[300,200,100,50]	mle	MinMax	0.1

Layer Configuration (LC), Principal Component Analysis (PCA).

**Table 32:** Performance on difference batch size.

Dataset	Epoch = 20		
	Batch Size = 1	Batch Size = 10	Batch Size = 20
Heart	84%	84%	84%
Virus	99%	99%	99%
Sonar	87%	87%	84%
Peugeot_Target 14	98%	99%	98%
Peugeot_Target 15	97%	99%	99%
EnviroCar	0.99	0.99	0.99
AQI	0.63	0.86	0.76

**Table 33:** Dropout effect on ANN.

Dataset	Dropout	
	0	0.1
Heart	84%	84%
Virus	99%	99%
Sonar	87%	83%
Peugeot_Target 14	99%	99%
Peugeot_Target 15	99%	99%
EnviroCar	0.99	0.99
AQI	0.86	0.82

**Table 34:** Training time for different values of the C parameter.

Dataset	C Parameter				
	0.01	0.1	1	10	100
Heart	<1 ms	<1 ms	<1 ms	<1 ms	<1 ms
Virus	100 ms	300 ms	500 ms	600 ms	600 ms
Sonar	<1 ms	<1 ms	<1 ms	<1 ms	<1 ms
Peugeot_Target 14	<1 ms	100 ms	200 ms	300 ms	400 ms
Peugeot_Target 15	<1 ms	100 ms	300 ms	300 ms	300 ms
EnviroCar	100 ms	100 ms	100 ms	100 ms	100 ms
AQI	<1 ms	<1 ms	<1 ms	<1 ms	300 ms
